# First Report of a Migratory Pest, the Fall Armyworm, *Spodoptera frugiperda* (JE Smith, 1797) (Lepidoptera, Noctuidae) from Bulgaria

**DOI:** 10.3390/insects16020134

**Published:** 2025-01-30

**Authors:** Szabolcs Szanyi, Marek Barta, Dimitar Velchev, Stoyan Beshkov, Stephen Mumford, Ivaylo Todorov, Antal Nagy, Zoltán Varga, Miklós Tóth, Teodora Toshova

**Affiliations:** 1 Institute of Plant Protection, Faculty of the Agricultural and Food Sciences and Environmental Management, University of Debrecen, Böszörményi út 138, 4032 Debrecen, Hungary; nagyanti@agr.unideb.hu; 2 Institute of Forest Ecology, Slovak Academy of Sciences, Akademická 2, 949 01 Nitra, Slovakia; marek.barta@savba.sk; 3 Maize Research Institute, Agricultural Academy, 5835 Knezha, Bulgaria; mitko_vel4ev@mail.bg; 4 National Museum of Natural History, Bulgarian Academy of Sciences, 1 Tsar Osvoboditel Blvd., 1000 Sofia, Bulgaria; stoyan.beshkov@gmail.com; 5 Independent Researcher, 4292 Dobri Dol, Bulgaria; steve.harvestbox@gmail.com; 6 Institute of Biodiversity and Ecosystem Research, Bulgarian Academy of Sciences, 1 Tsar Osvoboditel Blvd., 1000 Sofia, Bulgaria; i.toddorov@abv.bg (I.T.); teodora_toshova@yahoo.com (T.T.); 7 Department of Evolutionary Zoology and Human Biology, University of Debrecen, Egyetem tér 1, 4032 Debrecen, Hungary; varga.zoltan@science.unideb.hu; 8 Plant Protection Institute, CAR, HUN-REN, 1525 Budapest, Hungary; toth.miklos@atk.hun-ren.hu

**Keywords:** invasive species, maize, sex pheromone traps, light traps, food attractant, citizen science

## Abstract

The first occurrence of an invasive pest species, the fall armyworm (*Spodoptera frugiperda,* Lepidoptera: Noctuidae), in Bulgaria is reported. Different trapping methods (black light trap, traps baited with semiochemical lures, and food attractants) were used to study Noctuidae assemblages in Bulgaria in 2023. Two males of *S. frugiperda* were caught with sex pheromone traps in maize fields in Knezha, northern Bulgaria, in early November 2023. Additionally, three moths were attracted by black light traps (early November 2023) and one moth by a food lure applied on a tree trunk at the end of October 2023 in the municipalities of Parvomay and Chirpan, southern Bulgaria, respectively. The identification of *S. frugiperda* was confirmed using morphological and molecular methods.

## Simple Summary

The fall armyworm (*Spodoptera frugiperda* J.E. Smith, 1797) (Lepidoptera: Noctuidae) is an economically important polyphagous owlet moth that is native to the Americas. Over the last decade, this species has become a cosmopolitan pest due to introductions and spontaneous spread. Climate models indicate that the Mediterranean region offers optimal conditions for its establishment in Europe. However, the threatened area could expand significantly due to climate change. The fall armyworm has recently been reported in Spain, Cyprus, Greece, Portugal, Malta, and Romania. In this paper, we present the results of studies conducted in Bulgaria and report the first occurrence of this species in the country in samples collected in 2023.

## 1. Introduction

The fall armyworm (FAW), *Spodoptera frugiperda* (J.E. Smith, 1797) (Lepidoptera: Noctuidae), is native to and widely distributed in the tropical and sub-tropical regions of the Americas. FAW caterpillars are highly polyphagous, feeding on over 350 host plant species, and mainly cause damage to maize, rice, sorghum, cotton, and sugarcane [1]. The damage is mostly caused by the late instars, which feed on both the vegetative and generative parts of the host plants [2,3]. The damage may induce secondary infestations by saprotrophic and pathogenic ear mold fungi [4].

FAW has spread and colonized many countries around the world over the past decade. Although many regions of sub-Saharan Africa host year-round FAW populations, its potential for colonization in Mediterranean countries and seasonal migrations into the northern parts of Europe remains difficult to predict. Numerous studies have demonstrated the impact of climate change on the global distribution of pests with high adaptive ability, including FAW [5]. Despite its adaptability, FAW has been unable to colonize temperate regions [6]. In European countries, it has been reported in Spain (Canary Islands) (July 2020), Cyprus (January 2023), Greece (Kriti, January 2023; mainland, end of September–October), Portugal (Madeira 2023), Malta (September 2023), and Romania (end of October–beginning of November 2023) [7,8,9,10,11,12]. FAW is still classified as a quarantine pest in the EU, and it is also listed as an A1 quarantine pest by the EPPO. It is included in the list of European Commission pests (European Union 2019) due to its high risk of introduction and establishment. FAW’s resident populations are confined to relatively warm and moist areas since it cannot survive cold temperatures by entering diapause [13]. However, the fall armyworm poses a threat beyond its core area of distribution, even in temperate regions, because it is capable of undertaking long-distance seasonal migrations. 

## 2. Materials and Methods

The field sampling program using baited traps was carried out in maize fields of the Maize Research Institute in Knezha (Pleven Province, Bulgaria) (43.475416° N; 24.057916° E, 130 m a.s.l.) from 20th July to 8th November 2023. In the sampling area, arable lands are separated with forest strips of mixed tree species (*Robinia pseudoacacia* L., *Gleditsia triacanthos* L., *Ulmus minor* Mill., *Fraxinus* spp., *Prunus cerasifera* Ehrh., lindens, oaks, etc.). 

Traps were arranged in blocks, and each block contained one trap of each treatment. The distance between traps was 15 m, while between blocks it was at least 50 m (Table 1).

**Figure 1:**
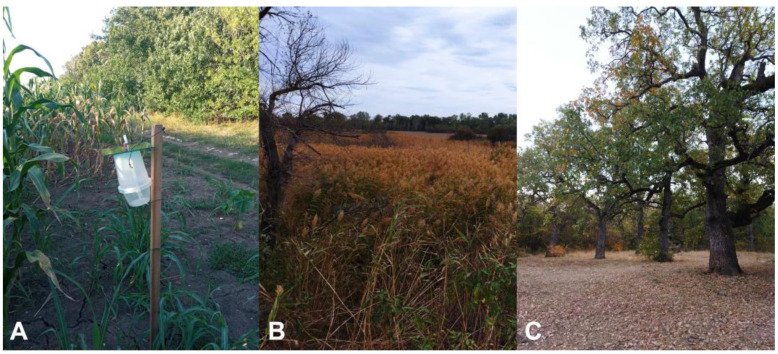
Habitats where FAW (*Spodoptera frugiperda*) adults were captured. (**A**) Knezha, (**B**) Dobri Dol, and (**C**) Chiprovska Koriya. Photos: T. Toshova (**A**) and S. Mumford (**B**,**C**).

CSALOMON^®^ VARL+ funnel traps (CSALOMON^®^, Plant Protection Institute, Centre for Agricultural Research, HUN-REN, Budapest; www.csalomontraps.com (accessed on 11 November 2024)) were used (Figure 1A). This trap has been developed for trapping of lepidopterans [14]. A piece (1 × 1 cm) of a household anti-moth insecticide strip (Chemotox^®^, Slouth, UK; active ingredient 15% dichlorvos) was placed into the container of the traps to kill captured insects.

Traps were baited with phenylacetaldehyde-based (FLO) or isoamyl-alcohol-based lures (SBLs), attracting Noctuidae species [15], or with *Spodoptera frugiperda* sex pheromone lures to test and compare their efficiency. The pheromone lure contained (Z)-9-tetradecenyl acetate plus (Z)-7-dodecenyl acetate, as described by Tumlinson et al. [16]. Unbaited traps were also used as controls. The test involved five replicates. 

Traps were attached to wooden poles at 1 m above the ground (Figure 1A). Traps were checked and samples were taken twice a week (a total of 32 times). Samples were stored deep-frozen at −18 °C in paper bags. At each sampling, the positions of the traps were rerandomized to eliminate the site effect. Lures were replaced after four-week periods (on 18th August, 15th September, and 13th October 2023).

### 2.1. Sampling with a Light Trap

A home-made light trap was used in a diverse landscape with vegetable garden, vineyard, fruit trees, and alfalfa field in Dobri Dol village, Parvomay Municipality, Plovdiv Province (42.131872° N, 25.310381° E; 117 m a.s.l.) for monitoring of Lepidoptera assemblages (Figure 1B). The sampling area is surrounded by arable lands, mainly wheat and maize fields. The light source was an OSRAM F15T8/350BL 15 W 18′′ preheat fluorescent Blacklight Lamp on a wooden box (30 cm in height). The light trap was placed at 1 m height. Samplings started after dusk (~19:00-21:00 CEST). Moths attracted to the light landed on molded pulp egg carton boxes placed below the trap. Species easy to identify at the species level were recorded, photographed, and released on site. The others (ca. 5–15% of moths sampled) were placed in killing jars containing 5 mL of acetone (Mavex, Rousse, Bulgaria) administered in cotton wool and kitchen paper. These were photographed later. All the pictures were uploaded to the citizen science platform iNaturalist (www.inaturalist.org, accessed on 11 January 2024), where community members made identifications and/or suggested corrections.

### 2.2. Sampling with Food Lure Applied on Tree Trunks

Food lure was used in a forest of the Chirpanskata Koriya Protected Area (Ancient Oak Forest), Chirpan Municipality, Stara Zagora Province (42.216350° N, 25.266484° E; 170 m a.s.l.), where the dominant tree species are *Quercus robur* L. mixed with *Q. pubescens* Wild, *Acer campestre* L., and *Carpinus betulus* L.) (Figure 1C). The area is bordered with coniferous tree belts (to north and west) and agricultural lands with wheat and maize fields (to south and east). The bait was prepared by mixing 3 kg sugar, 3 L red wine (Bear’s Blood, 10.5% Alc, Karnobat Winery, Bulgaria), and 2 L red wine vinegar (Kulinar, Vinprom Yambol, Bulgaria) and rotten fruits (banana and pear). A small amount of this mixture (100 mL) was applied on the bark of oaks (six trees) with a brush, which formed an about 20 × 40 cm strip at about 1.5m height.

Moths caught were identified at the species level and validated with dissection and examination of the male and female genitalia [17]. The genitalia were dissected following Fibiger [18], mounted on slides with Euparal, and photographed with a Zeiss stereo microscope Stemi 2000-C using a Canon 2000 digital camera.

### 2.3. Identification and Molecular Analysis by Sequencing COX1 Gene

Identification of the moths captured first occurred based on morphological characters. In this case, EPPO Diagnostic Protocol [19] was followed.

Genomic DNA was extracted from the legs and a distal part of the abdomen of the exsiccated moth voucher. The voucher (registered under the code of UEL-Lep-SF1) consists of a single male trapped by the VARL+ funnel trap with sex pheromone on 8 November 2023 at a sampling site in Knezha. The Qiagen DNeasy Blood & Tissue Kit (product ID: 69504, Qiagen N.V., Venlo, The Netherlands) was used for DNA extraction following the manufacturer’s protocol. A partial gene of cytochrome c oxidase subunit I (COX1) was amplified using the primer pair LCO (5′ GGTCAACAAATCATAAAGATATTGG 3′) and HCO (5′ TAAACTTCAGGGTGACCAAAAAATCA 3′) [20]. The PCR was performed in a 20-μL reaction volume using 2 μL of DNA extract in a PCR reaction mixture containing 10 pmol/µL of forward and reverse primers, 5× HOT FIREPol^®^ Blend Master Mix (Solis Biodyne, Tartu, Estonia), and deionized water of molecular grade (Pro injection, B. Braun, Melsungen, Germany). The PCR cycling profile included an initial denaturation at 95 °C for 15 min, followed by 40 cycles of denaturation at 95 °C for 30 s, annealing at 50 °C for 30 s, elongation at 72 °C for 90 s, and a final extension of 10 min at 72 °C. The PCR was performed in a Bio-Rad T100^TM^ Thermal Cycler (Bio-Rad Laboratories Inc., Hercules, CA, USA), and the PCR product was visualized on a 1% (*w*/*v*) TBE agarose gel stained with SimplySafe^TM^ stain (EURx, Gdansk, Poland). The target PCR fragment was purified using the QIAquick PCR Purification Kit (product ID: 28104, Qiagen N.V., Venlo, the Netherlands). Sanger sequencing was provided by SEQme Ltd. (www.seqme.eu, accessed on 11 January 2024, Dobříš, Czechia) on the ABI PRISM 3130 DNA analyzer. The sequence was manually checked in SnapGene^®^ Viewer, ver. 7.1.1 (GSL Biotech LLC, Boston, MA, USA) and compared using the BLASTN algorithm [21] against sequences deposited in the NCBI GenBank database.

## 3. Results

### 3.1. FAW Catches

**Figure 2:**
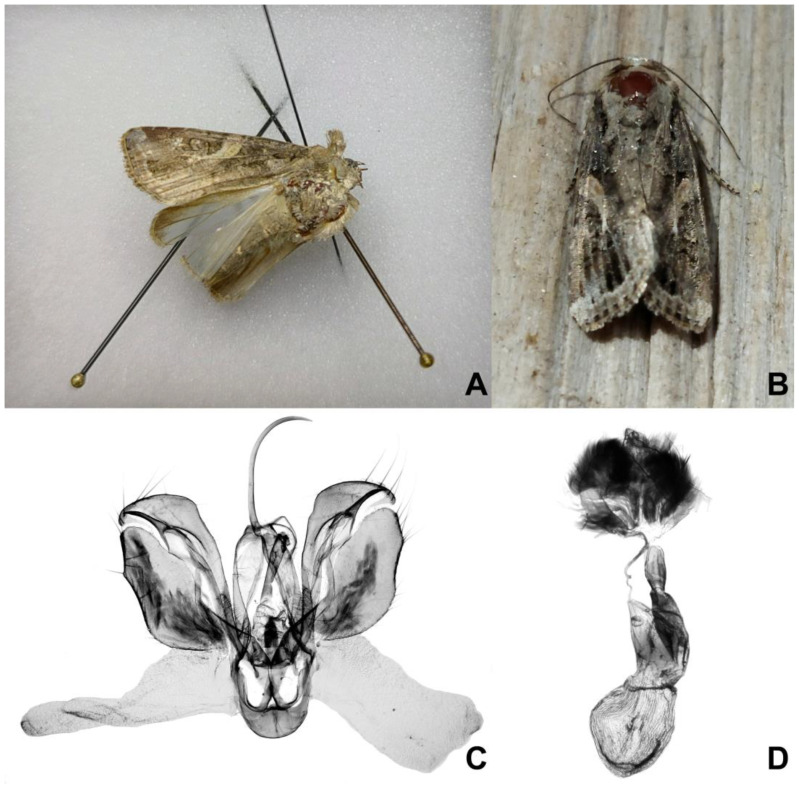
Male *Spodoptera frugiperda* from Knezha (6–8th November 2023) (**A**) and from Dobri Dol village (2nd November 2023) (**B**). Male genitalia of the moth caught in Dobri Dol (2nd November 2023) (**C**) and genitalia of female moth caught in Chirpanskata Koriya (30th October 2023). Photos: I. Todorov (**A**), S. Mumford (**B**), and S. Beshkov (**C**,**D**).

During the sampling procedures with traps baited with semiochemical lures, approximately 1600 noctuid moths were caught at the Maize Research Institute in Knezha. The results of the comparison of the tested lures will be published elsewhere. Based on their forewing patterns, two male noctuids caught with VARL+ traps baited with sex pheromone of FAW between 6 and 8th November 2023 were identified as *S. frugiperda*. Additional morphological and molecular analyses confirmed that these specimens were fall armyworms (Figure 2).

On 1st November in Dobri Dol, a blacklight trap caught five noctuid moths, among them one female *S. frugiperda*. It flew under relatively unfavorable conditions, with a temperature of 9 °C, 84% air humidity, atmospheric pressure of 997.5 hPa, and 0 km/h wind. One day later, on the 2nd of November, under similar conditions (temperature 11 °C, air humidity 98%, atmospheric pressure 993.4 hPa, and wind 0 km/h), the trap caught 70 noctuids, among them one female and one male of *S. frugiperda* (Figure 1B). Additionally, seven specimens of *Spodoptera exigua* (Hübner, 1808) were recorded on the same day. 

The food lure used in the native oak forest of Chirpanskata Koriya Protected Area attracted 80 moths belonging to the Noctuidae family on 30th October 2023. In the sample, there was one *S. frugiperda* female, and four other specimens belonged to *S. exigua*. The weather conditions were temperate, with a temperature of 15 °C, 72% air humidity, atmospheric pressure 1004.1 hPa, and 0 km/h wind.

### 3.2. Molecular Analysis

The DNA amplicon of the partial COX1 gene is 624 bp long. It was compared with data from GenBank, and the studied individual was identified as *S. frugiperda* with a 100% match with sequences in the database. The amplicon has been submitted to the GenBank database with accession number PP001710 (https://www.ncbi.nlm.nih.gov/nuccore/2638327097 (accessed on 11 November 2024)).

## 4. Discussion

Using different sampling methods, adult FAW specimens were caught in south and north Bulgaria, reaching the Danubian Plain. Data from Knezha (Pleven Province), Dobri Dol (Plovdiv Province), and Chirpanskata Koriya Protected Area (Stara Zagora Province) are the first records of the species in Bulgaria. Although only a few individuals were caught in different areas, the three methods (sex pheromone, blacklight traps, and food lure) were all successful and suitable for catching adult moths.

The migration simulation model of Wang et al. [22] predicted the risk, routes, and periods of the invasion from North Africa to Europe across the Mediterranean Sea in the period of 2016–2022. According to the model, the risk of the invasion is highest in Spain and Italy, followed by Turkey, France, Greece, and Portugal, with probabilities between 5.08% and 39.08%. The model predicted that “some moths would enter Greece via the Mediterranean Sea and then spread to western Turkey. A few FAW could reach as far as Romania after three consecutive nights of flight.” Recently, this species has already been recorded in three neighboring countries of Bulgaria—Turkey, Greece, and Romania. In Turkey, Pehlivan and Atakan [23] first registered FAW in Adana Province, and, later, Tonğa [24] reported its appearance in Şanlıurfa Province. From mainland Greece, it was first recorded in the autumn of 2023 [11]. In Romania, it was first reported by Cean et al. [8]. The first data from Bulgaria presented here suggest that the species is spreading north using the east Balkanian (Moesian) route. 

The invasion of *S. frugiperda* might have started from Egypt, the closest country in the Mediterranean region where FAW has spread since 2019 [25]. Between 3 and 16th November, 2023, one of the authors (S. Beshkov) conducted light trap samplings in the West Balkan in North Macedonia, Albania, Montenegro, and Bosnia and Herzegovina using different light sources, but not one *S. frugiperda* was collected; however, other migratory species were recorded. Additionally, there have not been any data on the FAW distributions from these countries, and this also suggests that FAW uses “the east Balkanian route” rather than invading across the Mediterranean Sea as the model of Wang et al. [22] suggested. In order to prove this hypothesis, extensive and systematic sampling should be conducted throughout the Balkan Peninsula in the near future, covering both the East and West Balkan routes.

Since FAW has great damage potential globally regarding major crops, information on the pest’s distribution, biology, and ecology is needed urgently. This information would support the risk assessments and development of appropriate management strategies at the regional, national, and international levels too. The recent data serve as a base for the improvement and readjustment of the migration simulation model [13]. Studies of newly discovered populations colonizing new areas enable us to adapt our knowledge to the local conditions.

Considering the actual FAW distribution map, monitoring efforts should be organized in the Carpathian Basin (in Hungary) and inner (Serbia) and western parts (Croatia and Slovenia) of the Balkan Peninsula in order to facilitate early detection.

## Figures and Tables

**Table 1:** Characteristics of the study sites in Knezha, Bulgaria, in 2023.

Block of Traps	Period of Trap Exposition	Crop, area (ha), Preceding Crop	Dates of Sowing and Harvesting of Maize	Coordinates, Altitude
I. *	20.07–23.10.	maize (lines), 1.2 ha, barley	28.04; 23.10.	43.479611° N; 24.069583° E 126 m a.s.l.
	23.10–08.11.	maize hybrid Kn-683, 7 ha, wheat	23.04; 05.10.	43.478194° N; 24.066222° E; 126 m a.s.l.
II. *	20.07–23.10.	maize (lines) 1.2 ha, barley	28.04; 23.10.	43.479472° N; 24.068833° E 126 m a.s.l.
	23.10–08.11.	maize hybrid Kn-683, 7 ha, wheat	23.04; 05.10.	43.476972° N; 24.062611° E; 128 m a.s.l.
III.	20.07–08.11.	maize hybrid Kn-683, 7 ha, wheat	23.04; 05.10.	43.476861° N; 24.062138° E; 129 m a.s.l.
IV.	20.07–08.11.	maize hybrid Kn-683, 7 ha, wheat	23.04; 05.10.	43.475416° N; 24.057916° E 130 m a.s.l.
V. *	20.07–15.09.	maize hybrid Kn-560, 4.5 ha, wheat	23.04; 15.09.	43.477888° N; 24.054583° E 141 m. a.s.l.
	15.09–08.11.	maize hybrid Kn-683, 7 ha, wheat	23.04; 05.10.	43.476194° N; 24.060027° E; 129 m a.s.l.

* Traps from 1st, 2nd, and 5th replications were moved to the field with a size of 7 ha after the maize was harvested. During the period of 23 October to 8 November 2023, all pheromone traps were in the 7 ha field.

## Data Availability

All relevant data are within the paper.
